# SKEWED MACROPHAGE POLARIZATION IN AGING SKELETAL MUSCLE

**DOI:** 10.1093/geroni/igz038.400

**Published:** 2019-11-08

**Authors:** Chang-Yi Cui, Riley Driscoll, Yulan Piao, Chee Chia, Myriam Gorospe, Luigi Ferrucci

**Affiliations:** 1 Laboratory of Genetics & Genomics, National Institute on Aging, Baltimore, Maryland, United States; 2 National Institute on Aging, Baltimore, Maryland, United States; 3 Translational Gerontology Branch, Intramural Research Program, National Institute on Aging, National Institutes of Health, Baltimore, Maryland, United States

## Abstract

Skeletal muscle aging is a major cause of disability and frailty in the elderly. The progressive impairment of skeletal muscle with aging was recently linked to a disequilibrium between damage and repair. Macrophages participate in muscle tissue repair first as pro-inflammatory M1 subtype and then as anti-inflammatory M2 subtype. However, information on the presence of macrophages in skeletal muscle is still sporadic and the effect of aging on macrophage phenotype remains unknown. In this study, we sought to characterize the polarization status of macrophages in human skeletal muscle at different ages. We found that most macrophages in human skeletal muscle are M2, and that this number increased with advancing age. On the contrary, M1 macrophages declined with aging, making the total number of macrophages invariant with older age. Notably, M2 macrophages co-localized with increasing intermuscular adipose tissue (IMAT) in aging skeletal muscle. Old BALB/c mice showed increased IMAT and regenerating myofibers in skeletal muscle, accompanied by elevated expression of adipocyte markers and M2 cytokines. Collectively, we report that polarization of macrophages to the major M2 subtype is associated with IMAT, and propose that increased M2 in aged skeletal muscle may reflect active repair of aging-associated muscle damage.

